# In-vitro and In-silico evaluation of the inhibitory effects of *Persea americana* leaf extract against calcium oxalate stones

**DOI:** 10.1007/s00240-025-01791-4

**Published:** 2025-06-30

**Authors:** Emel Akyol, Merve Danisman, Mualla Oner

**Affiliations:** 1 Department of Chemical Engineering, Yildiz Technical University, Davutpasa Campus, Esenler, Istanbul 34210 Turkey; 2 Department of Chemistry and Chemical Processing Technologies, Bayramic Vocational School, Çanakkale 18 Mart University, Çanakkale, 17700 Turkey

**Keywords:** *Persea americana* extract (PAE), Renal calculi, SwissADME, Inhibitor, Calcium oxalate crystallization

## Abstract

This study investigated the effects of *Persea americana* extract (PAE) as a potential inhibitor of calcium oxalate monohydrate growth using both in-vitro and in-silico methods with spontaneous crystallization to evaluate its effectiveness in preventing kidney stones. X-ray diffraction was used to characterize the morphology of calcium oxalate (CaOx) crystals, while scanning electron microscopy (SEM) was used to determine their particle size and crystal formation patterns. SwissADME (Absorption, Distribution, Metabolism, Excretion) computational analysis predicted the biological activity of the extract. The experimental results show that the inhibition of crystal growth increases with the amount of PAE in solution, and crystal growth was almost completely inhibited for 450 min at a concentration of 100 mL of PAE. The in vitro results also revealed that *Persea americana* leaf promoted the formation of calcium oxalate dihydrate (COD) crystals rather than monohydrate crystals. These findings on PAE's inhibitory effect on calcium oxalate crystallization suggest that natural additives could be valuable in treating kidney stone disease.

## Introduction

Stone formation in the kidney, bladder, or urinary tract (urolithiasis) is a biomineralization event that may occur in the human body, where substances such as uric acid and calcium, ordinarily present in urine, can crystallize and solidify into structures known as kidney stones or renal calculi [1–3]. The formation of urinary calculi involves a process of nucleation, growth, and aggregation of calcium oxalate, calcium phosphate, cysteine, and struvite crystals on epithelial cells at the renal papillae [4, 5]. According to Oner et al. (2022), this process is essentially driven by the supersaturation of urine with respect to the stone constituents; however, the crystallization kinetics are modulated by inhibitors and promoters of crystallization, which influence crystal growth, aggregation, and stone development [6]. Crystallization inhibitors play a critical role in the urinary stone system [7]. Kidney stones, also known as calculi, have been a common health problem since ancient times and adversely impact the quality of life of those affected [8]. Stone formation is still widespread today and affects approximately 12% of the population in developed countries [9]. Although there are different types, calcium oxalates (CaOx) are the most common component of over 70% of all human kidney stones [10]. CaOx crystals can be found in nature in three different morphologies: monoclinic calcium oxalate monohydrate (COM), tetragonal dihydrate (COD), and triclinic trihydrate (COT) [11]. COM is the thermodynamically stable phase of calcium oxalate, while the dihydrate and the trihydrate are metastable phases. COD is more easily expelled from the body [12]. The crystal nature depends on the crystal growth conditions such as temperature, supersaturation, solution nature, available free space for growth, and the presence of additives or impurities. Stone formation can be prevented by acid-rich urinary proteins in the human body under conditions of oversaturation. Stone formation inevitably occurs due to the absence of crystallization inhibitors. Substances can affect the crystallization and morphology of the CaOx crystal [13, 14]. Amino acids, citrate, and acid-rich proteins can inhibit CaOx crystallization [15].

Although various methods exist for treating stones formed in the kidney and urinary tract, not every technique is effective for every patient [16]. The treatment process is tailored to the location, size, number, and any additional health issues of the patient. While small stones (4–5 mm) can fall spontaneously by 40–50% with medical treatment, large stones (> 11 mm) require closer follow-up and monitoring. Dietary adjustments and increased fluid intake are recommended. In the treatment of stones, extracorporeal shock waves (ESWL) offer a non-invasive solution by breaking the stones, while percutaneous nephrolithotomy (PNL) removes the stones by entering the kidney through a small entrance. With ureteroscopy, stones are removed or crushed by inserting a scope into the urinary tract. Open surgery is preferred for large stones and obstruction, but the widespread use of ESWL and endourological methods has reduced the role of surgery [17–22].

Nowadays, immunomodulation, adaptogenic, and antimutagenic effects of herbal drugs have been clinically proven, which has led people to turn to nature against the high costs, side effects, and overuse of synthetic drugs. Medicinal plants are both a valuable and inexpensive resource, serving as a starting point for new drug discoveries. The World Health Organisation recommends the development and use of herbal and traditional medicines in both developed and developing countries, considering their cost-effectiveness and side effects [23–26]. The financial and other difficulties of surgical treatment necessitate improvements in drug treatments and the development of new therapies. Therefore, the inhibition of calculi growth has been an essential subject of many studies [16, 27, 28],

Many authors have reported the effects of a wide range of plant extracts on renal stone formation, including both in vitro studies and preclinical evaluations. *Persea americana*, commonly known as avocado or alligator pear, is a plant widely utilized in traditional medicine due to its diverse pharmacological properties. Various parts of the plant, including the leaves, seeds, and fruit, have been employed in the management of numerous ailments across different cultures. The medicinal properties of *Persea Americana* have been the focus of increasing scientific investigation, particularly concerning its potential antihypertensive, antimicrobial, anti-inflammatory, antidiabetic, and wound-healing effects. For instance, Odubanjo et al. (2016) investigated the combined use of avocado leaves and seeds in the treatment of hypertension, highlighting the plant’s potential cardiovascular benefits [29]. Makopa et al. (2020) further demonstrated the antibacterial, antifungal, and antidiabetic properties of avocado leaf extracts, suggesting a broad spectrum of therapeutic applications [30]. Rosa et al. (2024) examined the wound-healing effects of *Persea Americana*, while Retta et al. (2024) reported beneficial outcomes of its application in managing diabetic ulcers [31, 32]. These findings underscore the broad pharmacological utility of *Persea* americana and justify further exploration of its bioactive compounds. Numerous studies have also demonstrated that acidic compounds and certain minerals can effectively inhibit calcium oxalate (CaOx) crystallization, an essential factor in the pathogenesis of kidney stones [15, 33, 34]. Avocado leaves are known to be rich in organic acids, such as ascorbic and citric acid, as well as phytic acid and minerals like magnesium. These constituents have been previously associated with the prevention of CaOx crystal formation [18, 35]. Moreover, *Persea americana* leaf extract has demonstrated analgesic and anti-inflammatory effects, which may be beneficial in managing the painful symptoms associated with urolithiasis [36]. Several experimental studies have directly investigated the antiurolithiatic potential of *Persea americana* leaf extract. Wientarsih et al. (2012, 2014) and Sandhiutami et al. (2022) evaluated the effect of avocado leaf extract on calcium oxalate stone formation in white male rats [37–39]. Their findings indicated a reduction in stone burden, although the precise mechanism and optimal therapeutic dose remain unclear. Madyastuti et al. (2019) conducted a related study in vitro, combining *Persea americana* with Orthosiphon aristatus (cat’s whisker), and reported a positive effect on the dissolution of CaOx crystals [40]. Additionally, there is growing interest in understanding the phytochemical profile of *Persea americana.* The plant is rich in diverse secondary metabolites with therapeutic potential. Purwono et al. (2024) performed an in silico analysis of specific phytoconstituents, including oxoassoanine, ellipticine, acronycine, and quercetin, which are found in avocado leaves [41]. Meanwhile, Olufemi et al. (2024) conducted phytochemical profiling to evaluate the antioxidant potential of *Persea* americana leaves and seeds [42]. Despite these promising findings, the scope of in silico studies on this plant has been limited, and few have focused explicitly on its organic acids or their mechanisms in inhibiting CaOx crystallization. Although prior studies have indicated that *Persea* americana leaf extract may influence kidney stone formation, the exact therapeutic mechanisms, effective dosage, and its impact on CaOx crystal growth and morphology have not been conclusively determined. Furthermore, despite increasing interest in the health benefits of avocado leaves, comprehensive in silico and phytochemical analyses are lacking, and existing studies have focused on a narrow range of ligands.

Given the urgent need for safer and more effective alternatives to conventional stone treatment, and considering the promising preliminary evidence on the antiurolithiatic properties of *Persea americana*, this study aims to investigate the effects of avocado leaf extract on the kinetics and morphology of calcium oxalate monohydrate crystallization using both in vitro and in silico approaches. Additionally, the precise mechanism of action between avocado leaf extract and calcium oxalate crystals requires further clarification. Therefore, a comprehensive understanding of the extract's effect on kidney stones necessitates the detailed examination of its influence on calcium oxalate crystallization processes and associated kinetics. The in silico component specifically focuses on organic acids known to prevent calcium oxalate crystallization, such as ascorbic acid [43], phytic acid [44], and citric acid [45]. These compounds, despite their therapeutic relevance, have not yet been widely examined in computational models of CaOx inhibition. By integrating in vitro crystallization assays with molecular docking studies, this research seeks to advance our understanding of the mechanisms by which *Persea* americana leaf extract may contribute to the prevention or management of kidney stone disease. Based on these considerations, our investigation aims to evaluate the effects of *Persea americana* leaf extracts on both the kinetics and morphological characteristics of calcium oxalate monohydrate crystals in vitro, addressing the significant clinical challenges posed by urinary and kidney stone disorders.

## Materials and methods

*Persea americana* leaves were purchased from an authentic herb supplier in Istanbul's local market. Ten grams of dried *Persea americana* leaves were added to 100 ml of boiling distilled water and boiled for 15 min. The mixture was first allowed to cool, then coarsely filtered using Whatman No.1 filter paper to remove particulate matter. The resulting extract was subsequently passed through a sterile Millipore filter with a 0.22 μm pore size to remove any remaining fine particulates and ensure sterility before use in in vitro assays. The PAE extract was kept as a stock solution at −20 °C until use. The composition was determined using the AOAC method 986.13 for organic acid content. The AOAC methods provide detailed procedures and guidelines for accurate and reliable measurement of various substances, including food, pharmaceuticals, beverages, cosmetics, and dietary supplements. These methods are widely used in laboratories, regulatory agencies, and industry to ensure consistency and comparability of analytical results. Mineral content analysis was performed using AOAC Method 966.16 at the accredited Gozlem Food Control Laboratory Directorate. The chemical analysis of *Persea americana* leaf Extract is presented in Table 1. We calculated the individual ADME properties of the organic acid content using the Swiss ADME program (www.swissadme.ch) to understand their behavior. The results of each molecular property of citric, ascorbic, and phytic acids are given in Table 2.

**Table 1:** The chemical composition of *Persea americana* leaves extract

Minerals	Mineral composition (mg/L)	Organic acids	Organic acid compositions (mg/L)
Calcium (Ca)	78.00	Citric	414.00
Magnesium (Mg)	49.10	Ascorbic	52.70
Sodium (Na)	5.66	Phytic	68.10
Zinc (Zn)	0.82		
Potassium (K)	223.00		
Copper (Cu)	6.55		
Iron (Fe)	14.59

**Table 2:** Comprehensive analysis of organic acids’ physicochemical properties

Name	Ascorbic Acid	Citric Acid	Phytic acid
2D Structure			
3D Structure			
Molecular mass	176.12 g/mol	192.12 g/mol	660.04 g/mol
Molecular formula	C6H8O6	C6H8O7	C6H18O24P6
Molecule class	Vitamin, Antioxidant	Citrates, Antioxidant	Inositol Phosphates, Antioxidant
H-bond acceptor	6	7	24
H-bond donor	4	4	12
Rotatable bond	2	5	12
Heavy atoms	12	13	36
Molar Refractivity	35,12	37,47	101,27
TPSA	107.22 Å^2^	132.13 Å^2^	459.42 Å^2^

The effects of a *Persea americana* leaf extract on calcium oxalate monohydrate crystallization have been examined using the spontaneous crystallization method. Crystal growth experiments were conducted in a water-jacketed Pyrex glass vessel with a 1 L capacity at 37 °C. Calcium chloride (CaCl_2_) (J. T. Baker), sodium oxalate (Na_2_C_2_O_4_) (J. T. Baker), and *Persea americana* leaves were used as primary materials. Calcium oxalate calculi were grown from aqueous CaCl_2_-Na_2_C_2_O_4_ solutions in the absence and presence of PAE. The in vitro inhibitory effect of extracts of *Persea americana* leaves on the growth of COM in supersaturated solution was found by monitoring the decrease in [Ca^2+^] as a function of time. The crystallization process, which involves a progressive decrease in calcium ion activity over time, was tracked and measured using a Radiometer Impulsomat (PHM290) device equipped with a calcium ion-selective electrode (Ca-ISE, Radiometer ISE-K-CA). To corroborate the calcium ion measurements, the liquid phase was also analyzed for calcium content using atomic absorption spectroscopy (AAS) with a Perkin Elmer AAnalyst 200 instrument. The findings from AAS were in agreement with those obtained via the Ca-ISE, confirming that the reduction in soluble calcium levels corresponded to the formation of calcium oxalate crystals.

The effect of an additive can be measured by comparing the crystallization rate of the pure solution *(R*_*0*_) with the crystallization rate in the presence of the additive *(R*_*i*_) at the same concentration and temperature. Each rate determination was based on a minimum of three independent replicates, yielding a reproducibility within a 4–5% margin. Details of the experimental procedure have been reported in our previous works [11, 15]. The crystallite morphology and sizes of CaOx samples were analyzed by scanning electron microscopy (SEM, JEOL JSM 6335 F) and X-ray powder diffraction (Panalytical X’pert Pro PW 3040/60). X-ray diffraction analysis of the COM samples was carried out by a diffractometer with nickel-filtered Cu K*α* (*λ* = 1.54°A) as a radiation source and at a 2*θ* scan speed of 1°/min at 30 kV and 20 mA.

## Results and discussions

The crystallization rate of CaOx has been investigated in the presence and absence of PAE at 37 °C. The effect of the extract on crystal growth inhibition was determined by comparing the growth rate of CaOx crystals in the absence (*R*_*0*_*,*) and the presence of the PAE (*Ri)*. *R*_*0*_/*Ri* ratios were used to evaluate the success of PAE extract as an inhibitor.

**Fig. 1:**
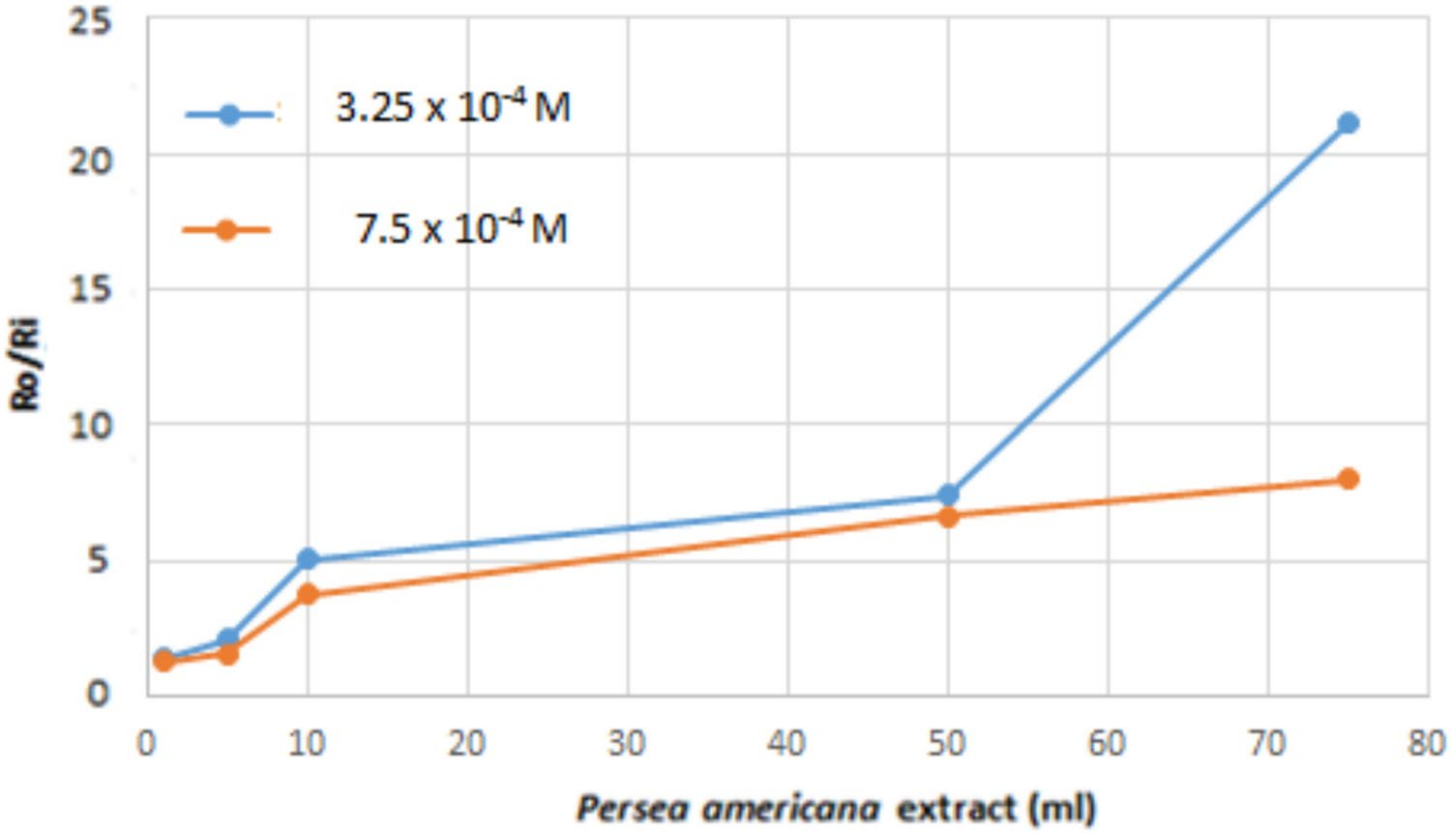
Effect of PAE on calcium oxalate crystallization at two different initial supersaturations

**Fig. 2:**
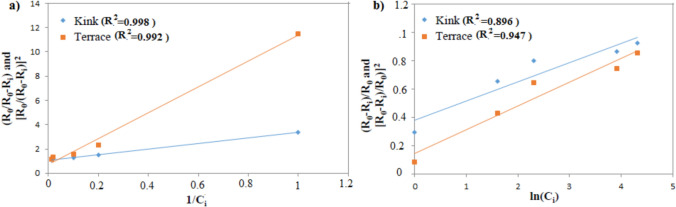
Calcium oxalate growth kinetics in the presence of PAE. Data is plotted according to the dependences predicted by (**a**) Langmuir model, (**b**) Temkin model

Figure 1 shows the effect of PAE amount on the crystallization rate. The higher *R*_*0*_*/Ri* values correspond to better inhibition. The experimental results show that *R*_*0*_*/R*_*i*_ ratios increase with increasing PAE amount. The crystal growth was almost completely inhibited for 450 min at 100 mL of PAE. The herbal extract exhibited more inhibitory effects at low supersaturation, as stone formation is often associated with increased supersaturation [17]. The addition of certain substances, known as additives, can affect the growth kinetics of crystals by modifying the properties of the growth medium [46, 47]. These modifications can include altering the phase equilibria, speciation in the medium, solvation and desolvation energies, and the mobility of growth units. As a result, the step velocity and growth rate of the crystals may increase or decrease. Predicting the effects of additives is challenging due to the need for a comprehensive understanding of solution and surface chemistry. However, it is possible to explain how some additives behave as stoppers by using simple physical models. Several studies have shown that organic materials inhibit crystallization kinetics by adsorbing onto the surface of crystals and subsequently covering them [18, 34]. The adsorption mechanism was investigated by adopting the Langmuir and Temkin adsorption models. These models are generally applied to the growth of polygonal crystals in a solution. According to the literature [23], the inhibition of PAE on calcium oxalate crystallization can be explained by applying the following equation;

1 $$\text{Langmuir isotherm}:{\left(\frac{{R}_{0}}{{R}_{0}-{R}_{i}}\right)}^{n}={\alpha }^{-n}\left(1+\frac{1}{K{C}_{i}}\right)$$

2 $$\text{Temkin isotherm}: {\left(\frac{{R}_{0}-{R}_{i}}{{R}_{0}}\right)}^{n}=z{\alpha }^{-}\left(ln{C}_{0}+ln{C}_{i}\right)$$

where *K, C*_*0*_*, Z* are constants, and *C*_i_ is the amount of inhibitor in the solution. α is the additive effectiveness parameter, and while the exponent *n* = 1 shows the case at which additive adsorption occurs at kinks in step edges as in the Kubota-Mullin model, *n* = 2 represents adsorption on the surface terrace as in the Cabrera-Vermilyea model [19, 20, 48]. Although Langmuir and Temkin’s models gave a good fit, the Langmuir model does a better data fit (Fig. 2). According to Fig. 2, PAE adsorbs preferentially on the terraces rather than at the kinks.

**Fig. 3:**
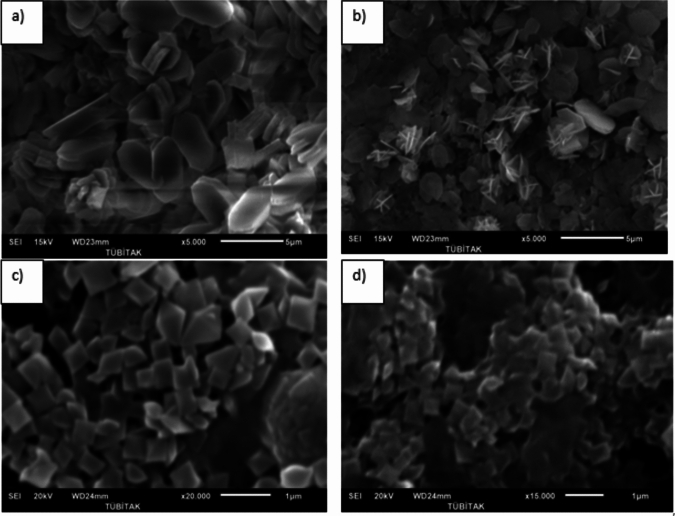
SEM of calcium oxalate crystals (**a**) control, (**b**) 10 mL PAE, (**c**) 50 mL PAE, (**d**) 75 mL PAE

The effect of PAE on the particle size and morphology of calcium oxalate crystals was investigated using scanning electron microscopy (SEM). The dimensions of at least 100 crystals in each sample were measured using the SEM. The average values representing these dimensions are presented in Table 3. The presence of herbal extracts in supersaturated solutions affected not only crystal size but also morphology (Fig. 3). From SEM images, monoclinic calcium oxalate monohydrate crystals were the major components in the absence of *Persea americana* leaf extract. Also, the crystals obtained in the presence of 10 ml of *Persea americana* leaves are in the COM structure. When 50 ml and 75 ml PAE are used, it is observed that all the crystals are within the COD structure. In addition, it was observed that the crystal sizes decreased with the increase of the *Persea americana* leaf extract additive. While the crystals with an average length of 7.92 μm were obtained in the absence of PAE, the length of the COM crystals was reduced to 4.80 μm in the presence of 10 ml of PAE. The results reveal that essentially all the crystals have exhibited typical tetragonal COD and aggregates following COD {1 1 0} penetration twinning law in the presence of 50 ml of PAE.

**Table 3:** The effects of PAE on CaOx crystallization

PAE (ml)				
	a (µm)	b (µm)	a (µm)	b (µm)
10	7.15(± 0.55)	4.71(± 0.43)	-	-
50	-	-	0.70(± 0.22)	0.62(± 0.19)
75	-	-	0.57(± 0.13)	0.53(± 0.12)

**Fig. 4:**
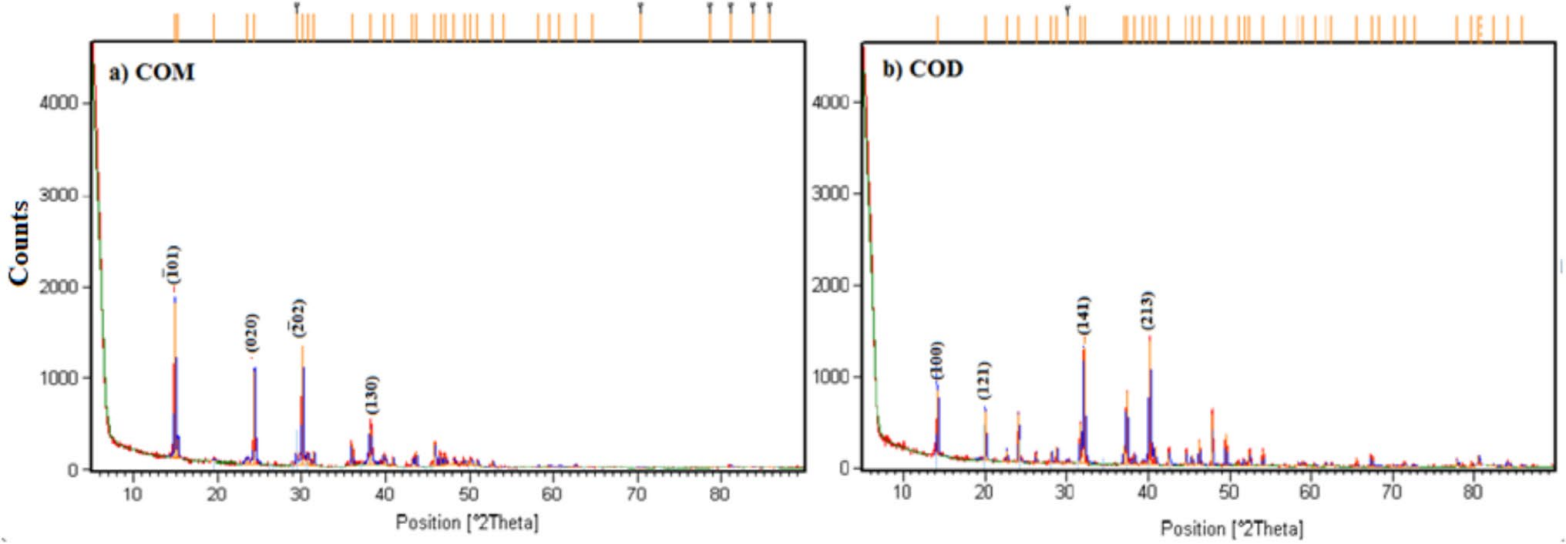
XRD powder patterns of crystals in the absence (**a**) and in the presence of 50 mL PAE (**b**)

Figure 4a and Fig. 4b show the differences between the X-ray diffraction patterns of crystal samples without PAE and in the presence of PAE. As revealed by XRD, the grown contact twinning crystals in the absence of PAE were identified as COM, and the XRD pattern of Fig. 4a matched with the JCPDS card number 20–231. The JCPDS card is a standardized data sheet that contains information about the crystalline structure of a material. X-ray diffraction (XRD) analysis (Fig. 4a) confirmed that the prismatic crystals grown were composed of calcium oxalate monohydrate (COM), which was further validated by comparison with the powder diffraction File 9-432JCPDS2000. The distinctive peaks of COM were observed at specific 2θ values, namely 14.931° (reflection (101)), 15.291° (reflection (110)), 24.371° (reflection (020)), 30.111° (reflection (202)), and 38.140° (reflection (130)). The intensity peaks in the XRD patterns of the COM powders closely matched the structural data described in the standards, although the intensity levels varied depending on the reaction conditions. The reflection peaks observed at 14.280°, 20.022°, 32.178°, and 40.152° confirmed the presence of COD (weddellite) in Fig. 4b. The diffraction peaks obtained in the PAE correlate well with the (hkl) indices of the COD phase (JCPDS card number 17–541). The prismatic calcium oxalate monohydrate (COM) crystals were eliminated, resulting in the formation of tetragonal calcium oxalate dihydrate (COD) crystals exhibiting bipyramidal morphology with distinct pyramidal end caps. It is important to note that COD crystals typically adopt a bipyramidal shape, which has shown a preference for the preferential growth of specific crystallographic faces. It is thought that the acidic proteins in *Persea americana* cause changes in the morphology of the crystal by affecting the structure of the growing crystals, and depending on the application method, they bind to the crystal surface and prevent the growth and aggregation of COM crystals and convert them into COD form. This result is consistent with previous studies, which have shown that organic acids such as citric acid, amino acids, glutamic acid, and ascorbic acid effectively prevent crystallization [23–25].

**Fig. 5:**
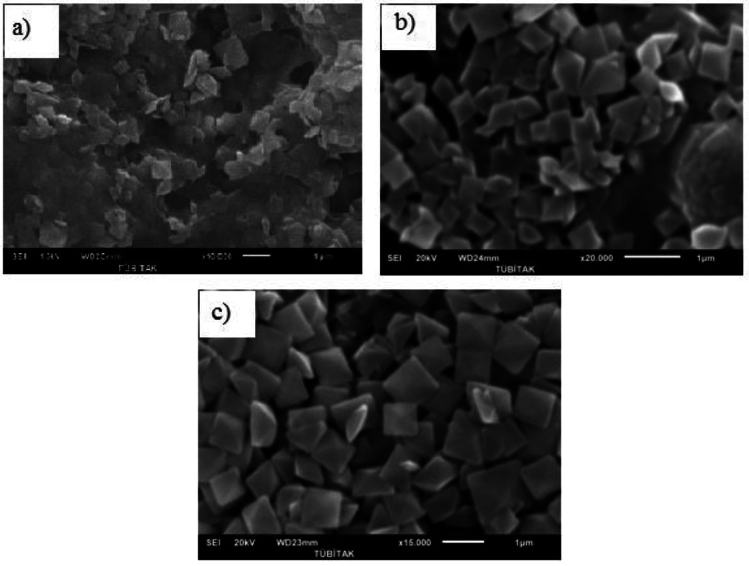
SEM images of calcium oxalate crystals with the addition of 75 ml PAE extract at different initial supersaturation; (**A**) 3.25 × 10^–4^ M, (**B**) 4.5 × 10^–4^ M, (**C**) 5.5 × 10^–4^ M

A standard experimental procedure was followed to examine the effect of initial supersaturation on calcium oxalate crystallization, and experiments were conducted under identical conditions. Experiments were carried out at initial supersaturation of 3.25 × 10^–4^ M, 4.5 × 10^–4^ M, 5.5 × 10^–4^ M and 6.5 × 10^–4^ M with 75 ml *Persea americana* leaf extract. SEM images of the samples obtained from the experiments are shown in Fig. 5.

In the SEM images obtained, it was observed that the calcium oxalate crystals formed from experiments conducted at different initial supersaturation were in the COD structure. In addition, the increase in the initial supersaturation led to an increase in the sizes of the formed crystals. By analyzing the SEM images, it was observed that the addition of 75 ml of *Persea americana* leaf extract during experiments at varying initial supersaturations resulted in the formation of calcium oxalate crystals with a COD structure.

## ADME and drug-likeness analysis

Here we will discuss how we predict ADME parameters and some pharmacokinetic properties of the molecules in silico. The SwissADME web server is one of the widely used tools to perform this prediction [26, 49, 50]. Oil compounds in the extract of *Persea americana* met the druglikeness according to Lipinski's rules using SwissADME. Table 4 highlights the significant differences in the predicted ADME properties of the tested compounds, which are outlined in more detail below.

**Table 4:** Pharmacokinetic properties and drug-likeness of organic acids

Organic acids			Ascorbic acid	Citric acid	Phytic acid
GI Absorption			High	Low	Low
BBB Permeation			No	No	No
Consensus* Log P*_*o/w*_			−1.28	−1.51	−6.77
P-gp substrate			No	No	Yes
*Log Kp* (skin permeation) cm/s			−8.54	−8.69	−17.63
*Log S (ESOL)*			0.23	0.38	3.34
Bioavailability			0.56	0.56	0.11
Synthetic accessibility			3.47	2.18	5.86
Inhibition of *Cytochrome P450*		CYP1A2	No	No	No
		CYP2C19	No	No	No
		CYP2C9	No	No	No
		CYP2D6	No	No	No
		CYP3A4	No	No	No
Drug-likeness	*Lipinski*		Yes	Yes	No
	*Ghose*		No	No	No
	*Veber*		Yes	Yes	No
	*Egan*		Yes	No	No
	*Mugge*		No	No	No
Medicinal properties	*TPSA (Å*^*2*^*)*		107.22 Å^2^	132.13 Å^2^	459.42 Å^2^
	*PAINS (alert)*		0	0	0
	*Lead likeness*		No	No	No

*Log S* provides information about water solubility. The ideal range is usually between −0.5 and 5. Very high *LogP* values ​​(> 5) may indicate low solubility and poor bioavailability. Consensus *Log P*_*o/w*_ is the average value of the n-octanol/water partition coefficient (*Log P* or *Log Po/w*) of a compound as estimated by SwissADME. This value represents the balance between the hydrophobicity (oil solubility) and hydrophilicity (water solubility) of the compound. The Consensus *Log P*_*o/w*_ value of all three organic acids being less than 0 indicates their solubility in water. A high GI Absorption value is preferred, as this is important for oral bioavailability. Blood–Brain Barrier (BBB permeation): It may not be necessary to pass through the brain barrier in the treatment of kidney stones, so being"No"does not cause a problem*. P-gp Substrate*: Whether a drug is a *P-glycoprotein substrate* or not is important for its cellular excretion. Being"No"may be advantageous in terms of intracellular accumulation. Inhibition or metabolism with enzymes such as CYP3A4, CYP2D6, and CYP2C9 should be checked. If it is a strong inhibitor, attention should be paid to potential drug interactions. Lipinski's 5 rule (Molecular weight < 500 Da, LogP < 5, H-bond donor < 10, H-bond acceptor < 5) must be met. PAINS (Pan-Assay Interference Compounds): If positive, the compound may be non-specific and cause false-positive results. According to Table 4, ascorbic acid and citric acid exhibited favourable oral bioavailability and drug similarity profiles, whereas phytic acid demonstrated low absorption and failed to meet most of the criteria for drug similarity due to its high polarity and low bioavailability. Nevertheless, phytic acid exhibited strong in vitro activity, likely due to its negative charge, which enables calcium binding and the inhibition of calcium oxalate crystallisation. These findings suggest that while ascorbic and citric acids may exert systemic effects, the activity of phytic acid may be due more to local interactions in the urinary tract. Overall, the ADME data, together with the in vitro results, suggest that different delivery strategies may be required to optimise the therapeutic potential of each compound.

## Conclusion

In conclusion, the extract of *Persea americana* leaves demonstrates a strong inhibitory effect on calcium oxalate crystal growth by adsorbing onto the crystal surface and effectively halting crystallization for 450 min at 100 mL of PAE in solution. Experiments conducted with and without PAE at varying initial supersaturations revealed that lower initial supersaturation led to decreased crystallization rates and crystal sizes. SEM and XRD analysis showed that PAE's presence transformed crystal morphology from contact twinning (COM) to the more easily eliminated tetragonal (COD) form. This inhibition likely stems from the high organic acid and magnesium content in *Persea americana* leaves. The inhibitory mechanism involves organic acids, such as phytic acid, adsorbing to active growth sites on crystal surfaces. Understanding these crystallization inhibitors and their mechanisms is vital for treating kidney and urinary stone diseases. The inhibitory properties of *Persea americana* leaves show promise in preventing urinary stones. Additionally, ADME analysis suggests that PAE extracts have potential as traditional medicine and nutraceutical products, though this requires validation through further experimental and clinical studies.

## Data Availability

No datasets were generated or analysed during the current study.
